# Effectiveness of Topical Oxygen Therapy in Gingivitis and Periodontitis: Clinical Case Reports and Review of the Literature

**DOI:** 10.3390/jcm13051451

**Published:** 2024-03-02

**Authors:** Amani M. Basudan, Irfan Abas, Marwa Y. Shaheen, Hamdan S. Alghamdi

**Affiliations:** 1 Department of Periodontics and Community Dentistry, College of Dentistry, King Saud University, P.O. Box 2455, Riyadh 11451, Saudi Arabia; mashaheen@ksu.edu.sa (M.Y.S.); dalghamdi@ksu.edu.sa (H.S.A.); 2 Department of Oral Implantology and Restorative Dentistry, Academy and Private Practice, Herenstraat 37, 1404 HC Bussum, The Netherlands; irfan.abas@gmail.com

**Keywords:** gingivitis, periodontitis, topical oxygen therapy, adjunct local therapy

## Abstract

Gingivitis and periodontitis are common oral pathological conditions. Several optional adjunctive local therapies are used clinically. While antibiotics and chlorhexidine are the most common agents of choice, their long-term use is associated with several adverse effects. Some of these include staining of teeth and restorations, cellular cytotoxicity and hypersensitivity. Topical oxygen therapy has been recently introduced and could be clinically capable of inhibiting plaque bacterial biofilm growth. Available as a mouthwash, toothpaste and oral gel, this formulation comprises cellulose, glycerol and sodium peroxoborate, and releases topical oxygen in a controlled manner. Moreover, it releases topical oxygen, in a controlled manner, and lactoferrin, which are capable of antibacterial action and stimulation of bone cells, respectively. The aim of this paper is to report a case of gingivitis and another case of periodontitis, both of which were successfully treated clinically with adjunctive local oxygen therapy (blue^®^m). Additionally, this paper aims to review the relevant literature in terms of adjunct topical or local therapies used in the treatment of gingivitis and periodontitis, in order to understand how local therapies are helpful and to know if local oxygen therapy is a suitable clinical alternative.

## 1. Introduction

Gingivitis is a common oral pathological condition, which affects people of all age groups and is characterized by inflammation of the gingiva, leading to bleeding on probing (BOP) [1]. Although the prevalence of gingivitis increases with increasing age, it is more common among adolescents during puberty and in pregnant women. Similarly, gingivitis is more prevalent in clinical scenarios wherein routine oral hygiene maintenance is challenged, such as ill-fitting restorations, presence of orthodontic braces and attachments and systemic conditions compromising oral hygiene practices [2]. Since retention of plaque bacterial biofilm on the tooth surface is the major cause of gingivitis, the primary goal of treatment focuses on plaque removal through mechanical debridement (scaling) and reinforcement of oral hygiene instructions [3]. In addition, several optional adjunctive local therapies are used clinically, and these include antibacterial agents, antioxidants and anti-inflammatory drugs, administered as either topical gels or mouthwashes [4]. Chlorhexidine is considered the current gold standard and is the most popular antibacterial chemical used for oral hygiene maintenance [5].

In addition to gingivitis, plaque accumulation subgingivally could also lead to periodontitis, which is an infectious condition characterized by advancing periodontal inflammation and progressive destruction of periodontal tissues [6]. Untreated dental biofilm induced gingivitis is considered as one of the prime risk factors for the development of inflammatory periodontal disease [7]. Periodontitis can lead to loss of the dentition, and ultimately masticatory and esthetic disability. In periodontitis, the disease process is a two-way problem wherein tissue destruction occurs not only due to inflammation, but also because of the host immune response. Especially, the presence of multiple bacteria, which exert their pathological effects through synergistic relationships, and within an impregnable mature plaque biofilm, would lead to diffusion of bacterial antigens through the periodontal junctional attachment and further activation of immune responses [8]. One of the most prevalent forms of periodontal disease is periodontitis, which sets in a vicious cycle of initiation through accumulation of subgingival plaque, followed by progressive bone loss and pocket formation that leads to even greater plaque accumulation [4,9,10].

In addition to mechanical therapies such as scaling and root planing (SRP), in certain clinical scenarios, patients with periodontitis benefit from locally administered antimicrobial adjuncts [4]. In most cases, local adjuncts are administered subgingivally at the time of SRP for sustained drug delivery, thereby helping eliminate microbial flora and alleviate inflammation [11]. While chlorhexidine is the preferred antibacterial agent of choice to be delivered locally for periodontitis treatment, its long-term use is associated with several adverse effects [5]. Some of these include the staining of teeth and restorations, cellular cytotoxicity and hypersensitivity [12,13,14]. Similar antibiotics such as tetracycline and metronidazole are not free from adverse effects when administered locally in the periodontal tissues [15].

In an effort to mitigate the untoward effects of locally delivered antibacterial and chemical adjuncts, periodontists and dental clinicians have been in search of a suitable alternative. Several novel compounds have been suggested, and even clinically tested for their benefits when administered locally in periodontal disease sites [4]. Oxygen therapy has been in use for almost a century and can be administered either systemically or topically. In addition to improving wound healing, oxygen administration is shown to promote angiogenesis, enhance chemotaxis of immune cells and inhibit inflammation and infection [16,17]. While hyperbaric oxygen is an effective route of systemic administration, local oxygen administration is still challenging in terms of obtaining a suitable carrier, which can deliver oxygen to the tissues [17]. Topical toothpaste and oral gel formulations, which release active oxygen molecules are not only capable of controlling plaque buildup, but are also effective against gingivitis and periodontitis, similar to results of subgingival scaling [7,18]. Recently, a research group comprising Dr. Peter Blijdorp developed a topical oxygen therapy formulation (blue^®^m), which was clinically capable of inhibiting plaque bacterial biofilm growth [19]. Available as a mouthwash, toothpaste and oral gel, this formulation comprises cellulose, glycerol and sodium peroxoborate, and releases topical oxygen in a controlled manner [20]. Additionally, it releases hydrogen peroxide and lactoferrin, which are capable of antibacterial action and stimulation of bone cells, respectively [21].

The aim of this paper is to report a case of gingivitis and another case of periodontitis, both of which were successfully treated clinically with adjunctive local oxygen therapy (blue^®^m). Additionally, this paper aims to review the relevant literature in terms of adjunct topical or local therapies used in the treatment of gingivitis and periodontitis, in order to understand how local therapies are helpful and to know if local oxygen therapy is a suitable clinical alternative.

## 2. Clinical Case Report 1—Topical Oxygen Therapy (blue^®^m) in the Treatment of Gingivitis

An otherwise healthy adult male patient reported to the dental clinic for comprehensive restorative treatment in the maxillary dentition, with fixed prosthodontics. Although the patient had started similar treatment in the mandibular teeth, he was not satisfied with the outcomes as his primary problem of persistent gum bleeding was not resolved. The patient gave a history of chronic bleeding from the gums, which persisted even after multiple dental treatments involving restorative and periodontal therapies, and regular oral hygiene maintenance.

**Figure 1:**
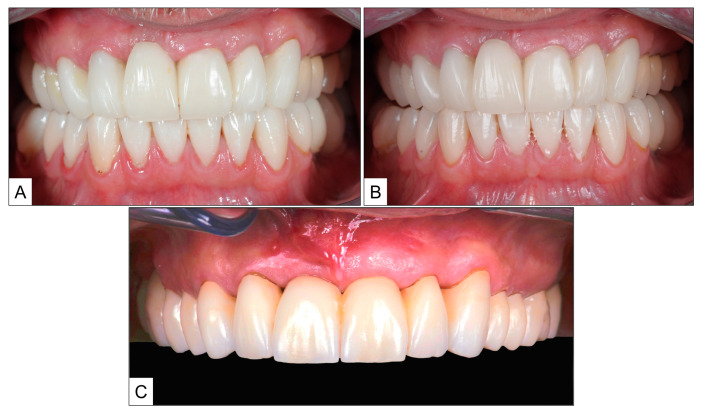
Topical oxygen therapy (blue^®^m) for treatment of gingivitis: (**A**) upon clinical presentation; (**B**) during recall visit 14 days after treatment with intraoral cleaning, scaling and topical oxygen therapy (blue^®^m toothpaste, mouthwash and gel); and (**C**) after restorative therapy and connective tissue augmentation labial to tooth #11.

Upon clinical examination, the patient had swollen gums with severe bleeding on probing. Nevertheless, there were no clinical signs of periodontitis, and the probing pocket depth (PPD) was no more than 3 mm at any particular site. We arrived at a clinical diagnosis of “dental biofilm induced gingivitis” (According to the World Workshop on Classification of Periodontal and Peri-implant Diseases and Conditions, 2017) [22,23]. Based on the above diagnosis, the patient was advised on treatment for the same before beginning restorative treatment in the maxillary arch. After giving consent, the patient underwent intraoral cleaning, scaling and oral prophylaxis. As a part of homecare, the patient was instructed to use a rotary electric toothbrush with blue^®^m toothpaste and to rinse the mouth twice a day for one minute with blue^®^m mouthwash. Additionally, the patient was advised to apply blue^®^m gel to the interproximal spaces using an interdental brush. When recalled after 14 days, all clinical signs of gingivitis had resolved and there was no bleeding on probing. The patient subsequently underwent restorative therapy of the maxillary dentition along with connective tissue augmentation of the gingival soft-tissue defect labial to tooth #11 (right maxillary central incisor) (Figure 1).

## 3. Clinical Case Report 2—Topical Oxygen Therapy (blue^®^m) in the Treatment of Periodontitis

**Figure 2:**
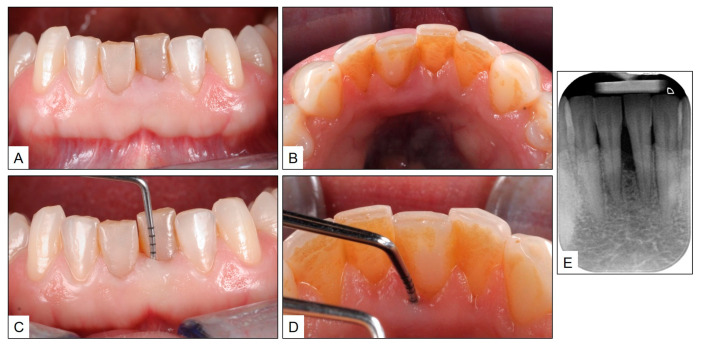
Pre-treatment clinical presentation with periodontitis in tooth #31: (**A**,**B**) inflamed gingival margin with slight grayish color; (**C**,**D**) probing pocket depth close to 7 mm on the labial and lingual aspects of the tooth; and (**E**) intraoral periapical radiograph showing close to 50% bone loss in the interdental area between teeth #31 and #41.

A 37-year-old healthy female patient reported to the dental clinic for a routine checkup. She was not a smoker and had no relevant social, medical or family history, other than the fact that her husband had lost all his teeth due to advanced periodontitis. Upon clinical examination, the patient had no dental caries and the periodontal examination was unremarkable except for the tooth #31 (left mandibular central incisor). The gingiva in relation to tooth #31 appeared slightly grayish in color, with a swollen gingival margin. Upon probing, the PPD was close to 7 mm on the labial and lingual aspects of the tooth and intraoral periapical radiograph revealed close to 50% bone loss in the interdental area between two mandibular central incisors. Based on the above clinical and radiographic findings, the case was diagnosed as periodontitis (Stage III, Grade B) in the mandibular central incisor area (Figure 2). It was classified as “Stage III” because of attachment loss in terms of a probing pocket depth of around 7 mm on the buccal and lingual aspects of the tooth, and in two adjacent lower anterior teeth, and “Grade B” considering an estimated 50% bone loss based on the patient’s age. This diagnosis was made in line with the classification system proposed by the “World Workshop on Classification of Periodontal and Peri-implant Diseases and Conditions, 2017” [22,23].

**Figure 3:**
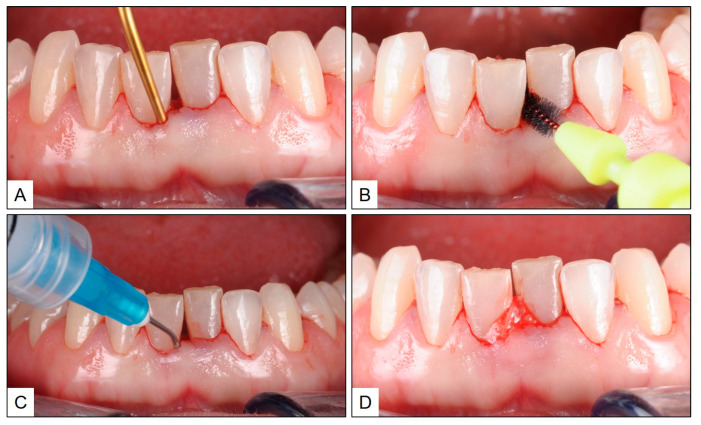
Treatment phase: (**A**) subgingival scaling and root planning in the mandibular central incisor area; (**B**) use of interdental brush in the interproximal space between teeth #31 and #41; (**C**) injection of the blue^®^m oral oxygen therapy gel in the mandibular central incisor area; and (**D**) leaving behind excess blue^®^m gel in situ supragingivally.

**Figure 4:**
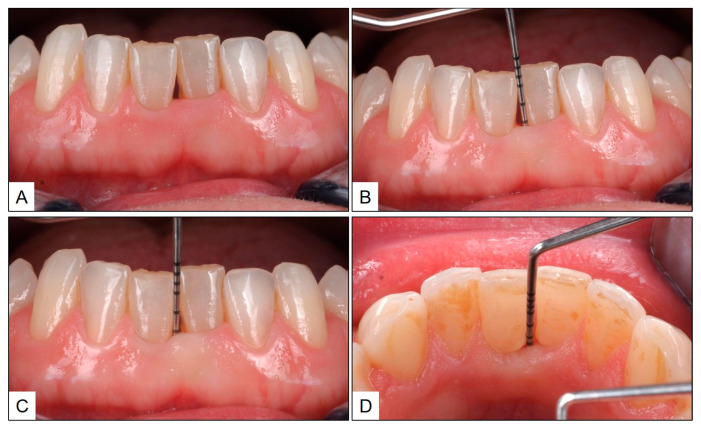
Six weeks post treatment: (**A**) healthy gingiva in the interdental area between teeth #31 and #41, with gingival recession and no clinical signs of inflammation; (**B**–**D**) probing pocket depth of up to 3 mm.

**Figure 5:**
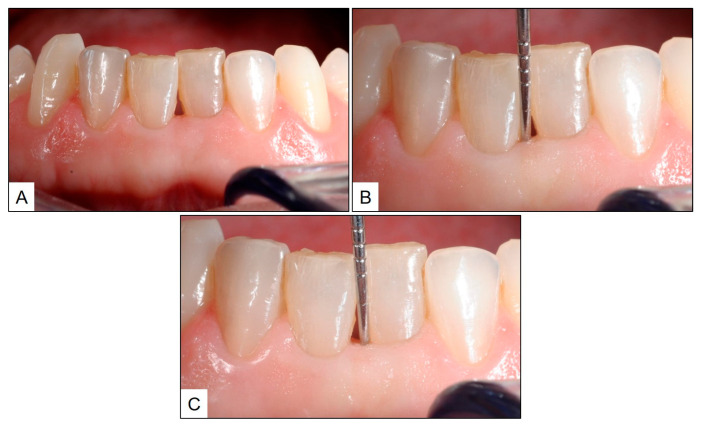
One-year post treatment: (**A**) healthy gingiva in the interdental area between teeth #31 and #41, with improving gingival recession and no inflammation; (**B**,**C**) probing pocket depth of up to 2 mm.

After obtaining consent, treatment was initiated with phase-1 periodontal therapy, including scaling and root planning (SRP) in the affected area, oral prophylaxis and hygiene reinforcement and the use of interdental brushes. Immediately after SRP, blue^®^m oral gel was injected into the gingival sulcus of the mandibular central incisors, for sustained local oxygen therapy, and any excess was left behind supragingivally (Figure 3). As a part of home care, the patient was advised to brush and rinse her mouth twice daily with blue^®^m toothpaste and mouthwash, respectively. In addition, the patient used blue^®^m oral gel with an interdental brush at the affected site, once in 12 h. The patient recall visit was planned six weeks after treatment and, at that time, the gingiva in the mandibular central incisor appeared healthy, without any clinical signs of inflammation. Although there was gingival recession in the interdental area between teeth #31 and #41, there was no bleeding on probing, and the PPD was only up to 3 mm (Figure 4). The patient was periodically recalled at three-month intervals, during which time the patient was reinforced to follow a home oral hygiene routine consisting of twice daily brushing and mouth rinsing, and interdental brushing once daily, with blue^®^m topical oxygen therapy-based products (toothpaste, mouthwash and oral gel). After one year, clinical examination revealed healthy gingiva between the mandibular central incisors, with PPD up to 2 mm and no bleeding on probing (Figure 5).

## 4. Systematic Review of the Literature—Methodology and Results

**Figure 6:**
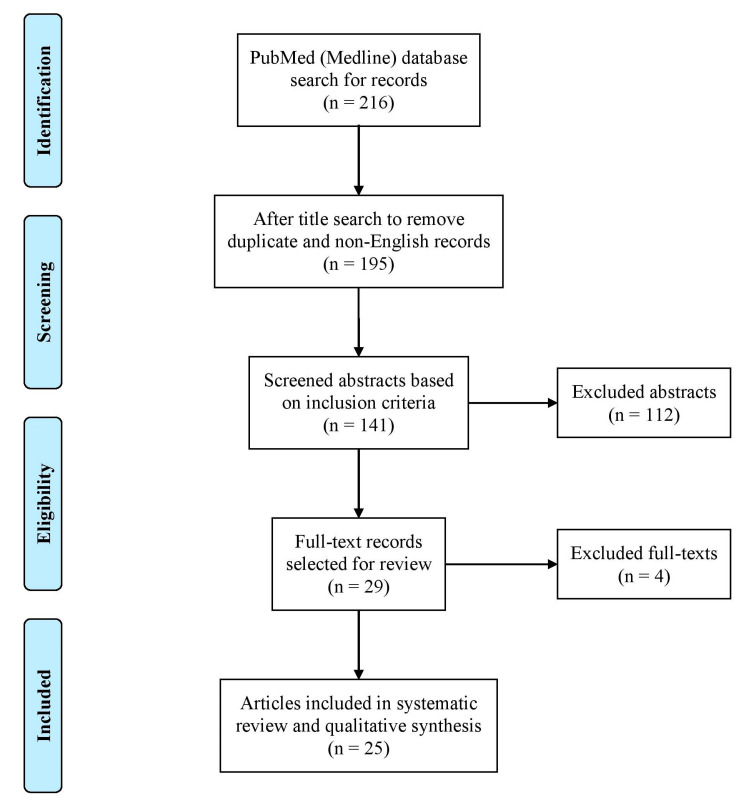
Flow chart describing the study selection process for the literature review.

A systematic review of the literature was conducted to know more about the currently used clinical adjunct topical or local therapies for the treatment of gingivitis and periodontitis. Within a period from 2010 to 2023, articles reporting the use of local adjuncts to mechanical debridement and SRP were searched on the PubMed (Medline) database. A combination of keywords, including but not limited to, “TOPICAL THERAPY”, “LOCAL THERAPY”, “LOCAL ADJUNCTS”, “GINGIVITIS”, “PERIODONTITIS”, “PERIODONTAL DISEASE”, “MECHANICAL DEBRIDEMENT” and “SCALING AND ROOT PLANING”, were used for the database search. An abstract review was conducted to exclude non-English articles, systematic reviews, case reports, clinical case series reporting less than 10 cases, editorial communications, in vivo animal experiments and in vitro studies. The flow chart describing the literature review process is shown in Figure 6. Twenty-nine clinical studies were identified for full-text review, out of which one study was excluded due to a pediatric study population [24], and three more studies were excluded due to the additional use of photodynamic therapy [25,26,27]. The nature of local adjunctive therapy used in the 25 reviewed studies, along with the reported improvements in clinical parameters and study outcomes, are detailed in Table 1.

## 5. Discussion

Based on a project to establish clinical practice guidelines for the treatment of different stages of periodontal disease, Sanz et al. [48] advocated an incremental approach to treatment. This included behavior modification, dental biofilm control through supragingival and subgingival scaling, non-surgical root planing, adjunctive therapies, surgical intervention and reinforcement of supportive personal oral hygiene practices [48]. The use of local therapy as an adjunct to mechanical debridement and SRP during the treatment of gingivitis and periodontitis is an interesting clinical strategy, as it helps in the delivery of the drug to the site of interest [4]. Local therapy may involve application of the drug or medicament either topically over the gingival surface or subgingivally inside the sulcus. While topical application may also be achieved through toothpastes and mouthwashes, subgingival administration predominantly requires gel-based formulations [46]. Local therapy not only helps to deliver a sustained dose of the drug to the periodontal tissues over a prolonged period, but also eliminates the risk of microbial resistance, especially with antibiotics and antibacterial drugs [30]. Difficulty of accessing deeper sections of pockets, furcation sites and root surface irregularities, and the inability to eliminate pathogenic flora from these areas is a major limitation of mechanical debridement and SRP, which can be overcome through local therapy [4]. In spite of convincing evidence in support of antibacterial agents like chlorhexidine and different antibiotics, there has been impetus towards identifying newer local adjuncts, which provide similar clinical benefits without the associated adverse effects [12,13,14,15].

Based on the literature review, several local adjuncts were reportedly used in addition to mechanical debridement and SRP. These included antibiotics, antibacterial agents, pharmacological drugs with a pleiotropic effects, plant-based compounds and vegetable extracts. While moxifloxacin, doxycycline, clarithromycin, metronidazole and satranidazole were the antibiotics reportedly used for local therapy, chlorhexidine and a combination of amine fluoride with stannous fluoride (AmF/SnF) were the reportedly used antibacterial agents. Pleiotropic effects of drugs like simvastatin, hyaluronic acid, alendronate, aspirin, ketoprofen, metformin, rosuvastatin and erythropoietin were also clinically evaluated for their use as a local therapy in gingivitis and periodontitis. The plant-based compounds and vegetable extracts reported include lycopene (carotenoid from tomatoes), ozonated olive oil, green tea catechin, curcumin (from turmeric) and grape seed extract. Except for AmF/SnF, which was administered only as a toothpaste or mouthwash, all other local adjuncts were based on gel formulations. Chlorhexidine was reportedly administered as both gels and mouthwashes.

Only clinical parameters such as a decrease in BOP, plaque index (PI), gingival index (GI) and gingival bleeding index (GBI), reduction in PPD and gain in clinical attachment level (CAL) were reported in the reviewed studies, after a post-treatment follow-up period ranging from 21 days to 6 months (Table 1). Considerable heterogeneity was observed in the reviewed studies in terms of reporting about smokers, non-smokers and patients with systemic illnesses.

In both the clinical cases reported in the present paper, local therapy was used as an adjunct to conventional treatment protocols, including oral prophylaxis, mechanical debridement, SRP and oral hygiene reinforcement. The only difference was in the use of a topical oxygen releasing formulation (blue^®^m), instead of other local adjuncts reported in the review. Moreover, the topical oxygen formulation was used as a toothpaste, mouthwash and oral gel, which was also applied subgingivally for sustained action. As mentioned earlier in this paper, the chemical composition of the topical oxygen formulation used in the reported cases enables the slow and sustained release of oxygen into the tissues from the site of administration. This is facilitated by the breakdown of peroxoborates into active peroxides, which are not only potent disinfectants, but are also a stable source of oxygen [19,20]. The presently used modality of topical therapy with blue^®^m formulations fulfills the important tenets of oxygen-based therapy, namely, to increase collagen formation and the metabolic activity of cells, promote growth factor release and angiogenesis and exert antibacterial and anti-inflammatory effects [17,46]. Yet another mechanism, which has been postulated in favor of topical oral oxygen therapy, is its small molecular size in comparison to other local therapeutic adjuncts such as antibiotics, chlorhexidine and pharmacological agents [17,19]. This enables the active oxygen molecule to penetrate the plaque biofilm and prevents adhesion of primary colonizer bacteria, thereby inhibiting the substrate for growth of planktonic bacteria in synergistic microbial colonies, similar to the ones causing gingivitis and periodontitis [49]. Lastly, based on the two reported cases, patients showed good compliance in the use of all types of topical oxygen therapy formulations (blue^®^m) and had no hypersensitivity reaction or adverse effects after subgingival administration.

Although the outcomes of the clinical cases reported herein are favorable and imply better clinical outcomes after gingivitis and periodontitis, they are limited by the small sample size, short follow-up period and the absence of suitable comparison groups. It would be our future endeavor to report a larger subset of clinical cases treated with topical oxygen-based therapy to provide formidable evidence in support of their role as a local adjunctive therapy for gingivitis and periodontitis. While the present clinical reports were focused mainly on evaluating the clinical outcomes of topical oxygen therapy, its effect on bone level gain could be included in the scope of future clinical trials. Similarly, the role of systemic illnesses and confounding factors such as smoking and parafunctional habits on the development and progression of gingivitis and periodontitis [50], and how they may be influenced by local oxygen-based therapy, would require further investigation.

## 6. Conclusions

Local adjunctive therapies to mechanical debridement and SRP are beneficial in the treatment of gingivitis and are an essential requisite in the management of periodontitis. The use of topical oxygen-based therapy blue^®^m formulations of toothpaste, mouthwash and oral gel yielded better clinical outcomes after treatment of patients with gingivitis and periodontitis. There was discernible resolution in signs of inflammation, marked decrease in BOP and reduction in PPD, similar to results of other local periodontal adjuncts reported in the literature. Based on the reported cases and outcomes of local therapy reviewed from the literature, it may be concluded that topical oral oxygen therapy (blue^®^m) is an effective adjunct to mechanical debridement and SRP in the management of gingivitis and periodontitis. Nevertheless, further large-scale, long-term comparative studies are required to design clinical protocols based on topical oral oxygen therapy.

## Figures and Tables

**Table 1:** Clinical studies reporting on adjunctive topical and local therapy with different materials for the treatment of gingivitis and periodontitis.

Author	Topical/Local Therapy Based on	Post Treatment Clinical Parameters	Outcomes/Conclusions
Pradeep et al. [28]	Simvastatin gel	Decrease in GBI—2.33 ± 0.80/Reduction in PPD—4.26 ± 1.59 mm/CAL gain—4.36 ± 1.92 mm	After 6 months, SRP with locally administered simvastatin gel gave significantly better clinical outcomes than SRP alone.
Guarnelli et al. [29]	Amine fluoride/Stannous fluoride (toothpaste and mouthrinse)	Decrease in PI *—Smoker (1.2)/Non-smoker (0.7)/Decrease in GI *—Smoker (0.2)/Non-smoker (0.3)/Reduction in PPD *—Smoker (0.5 mm)/Non-smoker (0.5 mm)	After 3 months, topical amine fluoride/stannous fluoride combination increased the efficacy of clinical outcomes compared with mechanical plaque control alone.
Flemmig et al. [30]	Moxifloxacin gel (0.125%, 0.4% and 1.25%)	Reduction in PPD—0.4% gel (1.5 ± 0.6 mm)/1.25% gel (1.2 ± 0.4 mm)/0.125% gel (1.1 ± 1.1 mm)	After 3 months, subgingival moxifloxacin 0.4% gel significantly reduced PPD compared with SRP alone.
Sapna and Vandana [31]	Hyaluronic acid gel (Gengigel^®^ applied topical and intrasulcular)	Decrease in PI—0.72 ± 0.38/Decrease in GI—0.83 ± 0.32/Decrease in GBI—5.53 ± 1.91	After 21 days, combined topical and intrasulcular hyaluronic acid gel administration significantly improved clinical outcomes and was equivalent to that of scaling alone.
Sharma and Pradeep [32]	Alendronate (1%) gel	Reduction in PPD—3.88 ± 1.39 mm/CAL gain—3.27 ± 1.11	After 6 months, alendronate 1% gel when used as an adjunct to SRP significantly improves clinical periodontal outcomes and results in better bone fill in areas of bone destruction due to aggressive periodontitis.
Funosas et al. [33]	NSAID gel (aspirin 1%, ketoprofen 1%, ketoprofen 2%)	Decrease in PI—aspirin 1% (1.04 ± 0.84)/ketoprofen 1% (0.93 ± 0.80)/ketoprofen 2% (1.09 ± 0.74)/Decrease in GI—aspirin 1% (1.42 ± 0.76)/ketoprofen 1% (1.21 ± 0.83)/ketoprofen 2% (1.23 ± 0.77)/Reduction in PPD—aspirin 1% (1.26 ± 0.13 mm)/ketoprofen 1% (1.02 ± 0.21 mm)/ketoprofen 2% (1.08 ± 0.28 mm)	After 30 days, aspirin 1% gel administered subgingivally after SRP was the most effective NSAID gel in improving clinical outcomes.
Tonetti et al. [34]	Doxycycline gel (slow release formulation)	Reduction in PPD—0.11 ± 0.03 mm	After 3 months, subgingival scaling and administration of doxycycline gel resulted in significant reduction of PPD compared with scaling alone.
Chandra et al. [35]	Lycopene gel		After 3 months, locally administered lycopene gel significantly reduced gingival bleeding and PPD, and increased CAL.
Madlena et al. [36]	Amine fluoride/Stannous fluoride (toothpaste and mouthrinse)	Decrease in PI—0.89 ± 0.15/Decrease in GI—1.05 ± 0.19	After 4 weeks, use of amine fluoride/stannous fluoride as a chemical adjunct to mechanical plaque control was significantly beneficial in patients with orthodontic braces.
El-Sayed et al. [37]	Hyaluronic acid gel (Gengigel^®^)	Decrease in BOP *—0.50/Reduction in PPD *—3.0 mm/CAL gain *—3.5 mm	After 6 months, locally delivered hyaluronic acid gel significantly improves clinical outcomes of periodontal surgery.
Agarwal et al. [6]	Clarithromycin (0.5%) gel	Decrease in PI—1.47 ± 0.18/Decrease in GI—0.74 ± 0.08/Decrease in GBI—0.91 ± 0.10/Reduction in PPD—2.53 ± 0.16 mm/CAL gain—1.52 ± 0.15	After 6 months, subgingival administration of clarithromycin 0.5% gel with SRP resulted in significantly enhanced clinical outcomes.
Patel et al. [38]	Ozonated olive oil	Decrease in PI—2.76 ± 0.16/Decrease in GI—2.17 ± 0.12/Decrease in GBI—4.15 ± 0.12	After 8 weeks, SRP in combination with topically applied ozonated olive oil improves clinical periodontal outcomes, similar to results of chlorhexidine.
Pradeep et al. [1]	Chlorhexidine gel + Metronidazole gel	Decrease in PI—2.41 ± 0.10/Decrease in GI—1.36 ± 0.08/Decrease in bacterial population—26.6 ± 0.34 (×10^4^ colonies)	After 6 months, topical application of a combination of chlorhexidine and metronidazole gel significantly improved clinical outcomes in gingivitis.
Pradeep et al. [11]	Metformin gel (0.5%, 1% and 1.5%)	Reduction in PPD—0.5% gel (2.97 ± 0.93 mm)/1% gel (4.0 ± 1.05 mm)/1.5% gel (3.8 ± 1.13 mm)/CAL gain—0.5% gel (2.23 ± 0.73 mm)/1% gel (3.83 ± 0.95 mm)/1.5% gel (3.6 ± 0.81 mm)	After 6 months, locally administered metformin gel (in differing concentrations) used as an adjunct with SRP significantly enhances periodontal clinical outcomes.
Chava and Vedula [39]	Green tea catechin gel	Decrease in GI—1.91 ± 0.20/Reduction in PPD—2.06 ± 0.07/CAL gain—2.1 ± 0.21	After 4 weeks, use of locally administered green tea catechin gel as an adjunct to SRP significantly enhances clinical periodontal outcomes.
Anitha et al. [40]	Curcumin extract	Reduction in PPD—2.97 ± 0.12/CAL gain—2.79 ± 0.21	After 30 days, locally administered curcumin extract as an adjunct to SRP significantly enhances clinical outcomes in comparison to chlorhexidine gel.
Priyanka et al. [41]	Satranidazole (3%) gel	Decrease in PI—0.18 ± 0.04/Decrease in GI—1.21 ± 0.06/Reduction in PPD—4.73 ± 0.33 mm/CAL gain—3.92 ± 0.29 mm	After 6 months, subgingivally administered satranidazole 3% gel as an adjunct with SRP significantly enhances periodontal clinical outcomes, in patients with type-2 diabetes.
Pradeep et al. [42]	Rosuvastatin (1.2%) gel	Decrease in GBI—3.71 ± 0.24/Reduction in PPD—4.04 ± 0.34 mm/CAL gain—4.2 ± 0.17 mm	After 6 months, subgingival delivery of rosuvastatin 1.2% gel as an adjunct with SRP resulted in significantly better clinical outcomes than SRP alone.
Pulikkotil and Nath [43]	Curcumin gel	Decrease in PI—0.42 ± 0.16/Decrease in GI—0.45 ± 0.31/Reduction in PPD—0.29 ± 0.67 mm	After 2 months, topically applied curcumin gel on gingivitis sites significantly improved clinical outcomes, equivalent to a combination of chlorhexidine and metronidazole gel, and better than application of chlorhexidine gel alone.
Kharaeva et al. [44]	Standardized fermented papaya gel		After 45 days, intragingival administration of papaya gel modulated the periodontal microenvironment resulting in synergistic antibacterial action with polymorphonuclear neutrophils, mediated through normalization of pro and anti-inflammatory cytokines.
Bergamaschi et al. [3]	Metronidazole (15%) gel	Reduction in PPD *—1.8 mm/CAL gain *—1.9 mm	After 6 months, topically applied metronidazole (15%) gel as an adjunct to periodontal debridement was equally effective as orally administered metronidazole (750 mg) and better than periodontal debridement alone.
Martin et al. [2]	Essential oil gel (with antioxidants phloretin + ferulic acid)	Decrease in PI—0.08 ± 0.07/Decrease in GI—0.14 ± 0.04/Reduction in PPD—0.04 ± 0.03 mm	After 5 weeks, although there was no statistically significant improvement in clinical parameters, topical antioxidant gel application on the gingiva helped reduce inflammation among orthodontic patients.
Rayyan et al. [45]	Grape seed extract gel	Decrease in PI—0.75 ± 0.71/Decrease in GI—0.85 ± 0.77/Reduction in PPD—0.65 ± 0.98 mm	After 6 months, subgingivally administered grape seed extract gel, as an adjunct to SRP for periodontitis, significantly improved gingival parameters only.
Al-Shammari et al. [46]	Hyaluronic acid gel (Gengigel^®^ applied subgingival)	Decrease in PI *—4.12/Decrease in GI *—4.11/Decrease in GBI *—4.04	After 12 weeks, subgingival administration of hyaluronic acid gel as an adjunct to SRP results in significant improvement of clinical parameters.
Aslroosta et al. [47]	Erythropoietin gel	Decrease in PI—1.42 ± 0.22/Decrease in GI—1.62 ± 0.17/Decrease in GBI—1.87 ± 0.15/Reduction in PPD—1.77 ± 0.19 mm/CAL gain—1.70 ± 0.89 mm	After 3 months, locally administered erythropoietin gel as an adjunct to SRP results in significant improvement of gingival and periodontal clinical parameters except PI.

GBI—Gingival bleeding index; PPD—Probing pocket depth; CAL—Clinical attachment level; SRP—Scaling and root planing; PI—Plaque index; GI—Gingival index; NSAID—Non-steroidal anti-inflammatory drug; BOP—Bleeding on probing; * Based on median values.

## Data Availability

All data are contained within the article.
